# Retraction: Role of globalziation defining the incidence of entrepreneurship

**DOI:** 10.1371/journal.pone.0346952

**Published:** 2026-04-14

**Authors:** The PLOS One Editors

The *PLOS One* Editors retract this article [1] due to concerns about:

- Non-compliance with PLOS policy on Authorship.
- Non-adherence to the journal’s first, second, third and fourth publication criteria.
- Insufficient detail of the specific datasets and sources of data used in this study to be reproducible.

NA and KH did not agree with the retraction. SA, SS, YAK, and SK either did not respond directly or could not be reached.
